# Effects of Immunotherapy on Quality-of-Life Outcomes in Patients with Gastroesophageal Cancers: A Meta-Analysis of Randomized Controlled Trials

**DOI:** 10.3390/healthcare12151496

**Published:** 2024-07-28

**Authors:** Kush Gupta, Arya Mariam Roy, Kristopher Attwood, Ryan David Nipp, Sarbajit Mukherjee

**Affiliations:** 1 Department of Internal Medicine, University of Massachusetts Chan Medical School-Baystate, Springfield, MA 01109, USA; kgupta1992@gmail.com; 2 Department of Hematology and Oncology, Roswell Park Comprehensive Cancer Center, Buffalo, NY 14203, USA; arya.roy@roswellpark.org (A.M.R.); kristopher.attwood@roswellpark.org (K.A.); 3 OU Health Stephenson Cancer Center, Oklahoma City, OK 73104, USA; ryan-nipp@ouhsc.edu

**Keywords:** esophageal cancer, gastric cancer, immunotherapy, chemotherapy, quality of life

## Abstract

Background: Immune checkpoint inhibitors (ICIs) have revolutionized cancer care, with increasing data demonstrating improved survival outcomes using ICIs among patients with advanced gastroesophageal cancer (GEC). ICIs are also associated with a lower incidence of grade ≥ 3 adverse events (AEs) compared to chemotherapy, suggesting that ICIs may have favorable effects on health-related quality of life (HRQoL). This meta-analysis sought to evaluate the effects of ICIs on the HRQoL of patients with advanced GEC. Methods: We conducted an online bibliographic search on Medline via PubMed using MeSH-based terms to retrieve randomized controlled trials (RCTs) that evaluated the effects of ICIs on HRQoL in patients with advanced GEC (we searched for all studies between 2018 and 2021). We included RCTs that incorporated ICIs as part of the intervention arm either as monotherapy (first or second line) or as a combination therapy (first-line) with another ICI or chemotherapy. We combined the HRQoL measures into a meta-analysis using standard random effects models, from which estimates of the average mean difference (MD) were obtained with 95% confidence intervals. We assessed the heterogeneity of the study outcomes using the Q and I^2^ statistics. Results: We identified 11 phase 3 RCTs that met the inclusion criteria, with a mean enrollment of 820 patients. Eight RCTs used an ICI plus chemotherapy combination in the intervention arm, three had ICIs as monotherapy, and one had doublet ICI therapy in the intervention arm. All RCTs used chemotherapy for the control arm. Collectively, the trials reported 37 HRQoL measures using five different HRQoL tools. The pooled analysis favored the intervention over the control arm in terms of the Functional Assessment of Cancer Therapy-Esophageal (FACT-E) scores [MD 2.7 (95% CI 0.1 to 5.3), *p* < 0.041]. In a subgroup analysis of eight RCTs comparing combination therapy with ICIs plus chemotherapy versus chemotherapy alone, the effect estimates favored the ICI arm regarding the FACT-E [MD 2.7 (95% CI 0.1 to 5.3), *p* < 0.041] and the EORTC QLQ-OES18 pain scale [MD −2.2 (95% CI −4.3 to −0.2), *p* < 0.030]. Likewise, the effect estimates favored the ICI monotherapy arm over the chemotherapy arm regarding the QLQ-STO22 hair loss subscale [MD −23.2 (95% CI −29.7 to −16.7), *p* < 0.001], QLQ-STO22 dysphagia subscale [MD 6.7 (95% CI 1.7 to 11.7), *p* = 0.009], EQ-5D pain scale [MD 6.9 (95% CI 2.9 to 10.9), *p* < 0.001], and QLQ-OES18 saliva subscale [MD 5.8 (95% CI 0.1 to 11.6), *p* = 0.046]. Conclusions: In this meta-analysis, we found that the inclusion of ICIs as a first-line treatment for advanced GEC yielded better HRQoL outcomes than chemotherapy alone. Further research on the impact of ICIs on HRQoL is needed, with increasing evidence that ICIs improve the survival outcomes in patients with advanced GEC.

## 1. Introduction

Gastroesophageal cancers (GECs), which encompass esophageal and gastric cancers, remain a leading cause of cancer-related morbidity and mortality globally, exerting a substantial healthcare and economic burden despite estimates suggesting a reduction in their incidence in recent decades [1,2]. Patients with GECs are often diagnosed at an advanced stage and thus have a poor prognosis, with a 5-year overall survival (OS) rate of 20–32% [3,4,5]. These patients experience a wide range of complications, including gastrointestinal bleeding, intestinal obstruction, and postoperative complications [6,7]. Evidence also suggests that advanced GEC patients may experience various physical and psychological symptoms and an impaired health-related quality of life (HRQoL) [8]. Notably, patients with GEC demonstrate a poor HRQoL compared to the general population, corresponding with their disease severity and symptom burden [9]. Importantly, pivotal randomized controlled trials (RCTs) have started incorporating HRQoL outcomes alongside traditional clinical endpoints, facilitating a more comprehensive understanding of the patient experience [10,11].

Systematic chemotherapy has long represented the standard first- and second-line treatment for patients with advanced GECs [12,13]. In recent years, immune checkpoint inhibitors (ICIs) targeting programmed cell death protein 1 (PD-1) or the PD-1 ligand (PD-L1) have emerged as a promising treatment option for patients with advanced GEC [14]. Previous RCTs demonstrated that ICIs can significantly prolong survival outcomes in patients with advanced GECs [15]. In the first-line setting, studies have shown non-inferior outcomes with ICI monotherapy compared to chemotherapy alone regarding survival outcomes, with a more favorable safety profile [16]. ICIs have a manageable safety profile, and their immune-related adverse events (AEs) differ from chemotherapy-related toxicities [17]. In addition, RCTs have demonstrated that ICIs are associated with a lower incidence of grade ≥ 3 AEs than chemotherapy [18], further favoring the benefit-to-risk profile of ICIs in GECs.

Most of the existing literature in GECs has focused on the survival benefits of ICIs over chemotherapy, with little emphasis on their impact on patients’ HRQoL. Standard chemotherapy regimens often have considerable toxicity, which can negatively impact patients’ HRQoL [19,20]. On the other hand, the favorable efficacy of ICIs and the lower incidence of grade ≥ 3 adverse effects (AEs) suggest a more favorable HRQoL in patients receiving ICIs. Recent subgroup analyses of these RCTs suggested a positive effect of ICIs on the HRQoL of patients with GEC [21]. The evidence is still limited by conflicting results and variability in the tools used to assess patients’ HRQoL [10,21,22,23].

In the current study, we sought to conduct a meta-analysis to pool data from pivotal RCTs and evaluate the impact of ICIs on the HRQoL of individuals with advanced GEC. Specifically, we compared the changes in the HRQoL of patients with advanced GEC who received ICIs versus those who received systemic chemotherapy. We hypothesized that those receiving ICIs would have favorable HRQoL profiles compared to those receiving chemotherapy. The findings from this work will help fill a needed gap regarding the impact of ICIs on HRQoL and help inform future efforts to understand patients’ care experience with GEC better.

## 2. Materials and Methods

### 2.1. Eligibility Criteria

We retrieved RCTs that fulfilled the following criteria: (1) studies that included adult patients (≥18 years old) with advanced, unresectable GECs; (2) studies that compared the outcomes of ICIs as monotherapy or in combination with chemotherapy versus chemotherapy alone; and (3) studies that reported the effect of the treatment groups on the patient-reported HRQoL outcomes. We searched for all studies between 2018 and 2021, and the patient population in each RCT is summarized in Table 1. There were no restrictions regarding the type of ICIs, country of publication, sample size, or duration of follow-up. We excluded case reports, reviews, preclinical and invitro studies, and studies that included patients with early-stage GECs.

### 2.2. Information Source, Search Strategy, and Selection Process

The present systematic review and meta-analysis were performed according to the Preferred Reporting Items for Systematic Reviews and Meta-Analyses (PRISMA) Statement and the guidelines for the meta-analysis of observational studies in epidemiology (MOOSE). PubMed, Embase, Scopus, and the Cochrane Library were searched for all relevant articles. The MeSH keywords are listed in Table 2.

Two independent reviewers screened the titles and abstracts of all identified studies for eligibility based on the predefined inclusion and exclusion criteria. The same two independent reviewers retrieved and reviewed the full-text articles of potentially eligible studies to confirm eligibility. Any discrepancies in study selection between the two reviewers were resolved through consensus or consulting with the senior investigator.

### 2.3. Data Collection Process

The extracted data included the summary characteristics of the included studies, the baseline characteristics of the patients, intervention characteristics, the changes in the HRQoL scores from baseline to the end of follow-up, and methodological data for assessing the risk of bias in the included studies. The summary of the retrieved studies included the year of publication, country, study design, population, sample size, type and dose of ICI regimen, chemotherapy regimens, and follow-up duration. We collected information on the baseline characteristics of patients, including their age, gender/sex, ECOG performance status, stage, histopathological subtype, PD-L1 expression, and number of metastases. The HRQoL tools included hte Functional Assessment of Cancer Therapy—Esophageal (FACT-E) and Gastric (FACT-G), EORTC Core Quality of Life questionnaire (EORTC QLQ-C30), EORTC Quality of Life Questionnaire—Oesophageal Cancer Module (EORTC QLQ-OES18), EORTC Quality of Life Questionnaire-Stomach (EORTC QLQ-STO22), and EuroQol-5 Dimension 3 (EQ-5D-3L) and five levels (EQ-5D-5L).

### 2.4. Statistical Analysis

We combined the HRQoL data across studies in a meta-analysis using standard random effects models, from which estimates of the average mean difference were obtained with 95% confidence intervals. If a given HRQoL measure was not reported for a given study, then that study was excluded from the corresponding analyses of the HRQoL measure. The Q and I2 statistics were obtained to assess the heterogeneity of the study outcomes. The Q statistic evaluates the homogeneity assumption (i.e., all studies have the same common mean difference). In contrast, the I2 statistic represents the amount of variability in the meta-analysis attributed to study heterogeneity. The publication bias was examined using funnel plots and the corresponding Egger’s test. There was no evidence of publication bias in our study. The mean differences for each HRQoL tool were presented visually using a forest plot. The primary analyses were performed using all available studies, while subgroup analyses were performed based on specific treatment combinations. All analyses were conducted in SAS v9.4 (Cary, NC, USA) at a significance level of 0.05.

## 3. Results

### 3.1. Search Results

**Figure 1:**
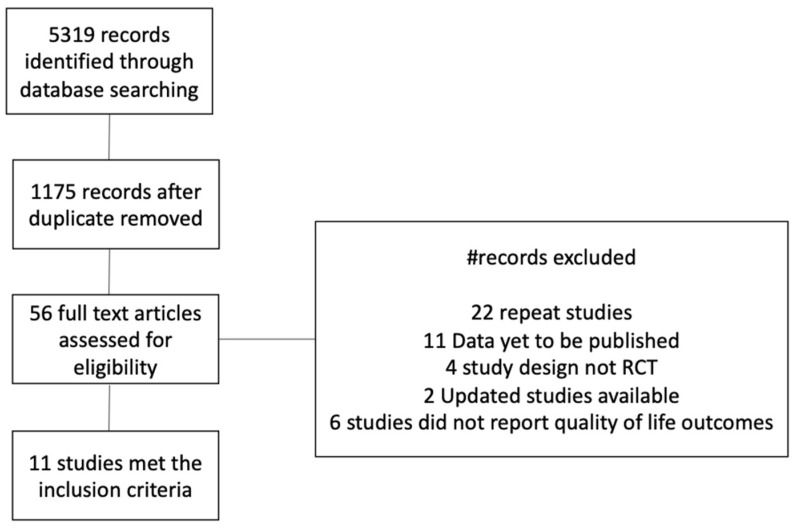
PRISMA flow diagram.

The bibliographic search yielded 5319 unique citations. Following the initial titles and abstracts screening, 56 citations were retained for full-text screening. Of these, 11 studies met the inclusion criteria. Thus, we included 11 studies in the current systematic review (Figure 1: PRISMA 2020 flow diagram).

### 3.2. Characteristics of the Included Studies

Among the 11 phase 3 RCTs included, five were double-blinded, two were single-blinded, and the remaining were open-label trials. Seven trials, such as CHECKMATE 648, KEYNOTE 061 ESCORT-1st, KEYNOTE 181, ATTRACTION-3, KEYNOTE 590, and ORIENT-15 [10,15,21,22,23,24,25], included patients with previously untreated, advanced, or metastatic esophageal squamous cell carcinoma or adenocarcinoma. Three trials, namely KEYNOTE 062, ORIENT-16, and ATTRACTION 4 [26,27,28], focused on patients with previously untreated, advanced gastric/gastroesophageal junction adenocarcinoma. In the CheckMate 649 trial [29], patients with previously untreated, unresectable, non-HER2-positive gastroesophageal adenocarcinoma were recruited and received either Nivolumab monotherapy, single-agent chemotherapy, or Nivolumab plus chemotherapy. Most studies evaluated pembrolizumab or nivolumab, and studies reported on 37 HRQoL tools (Table 1). Ipilimumab, Camrelizumab and Sintilimab were the other immunotherapy agents evaluated in the trials. The baseline characteristics of the included patients are summarized in Table 3.

**Figure 2:**
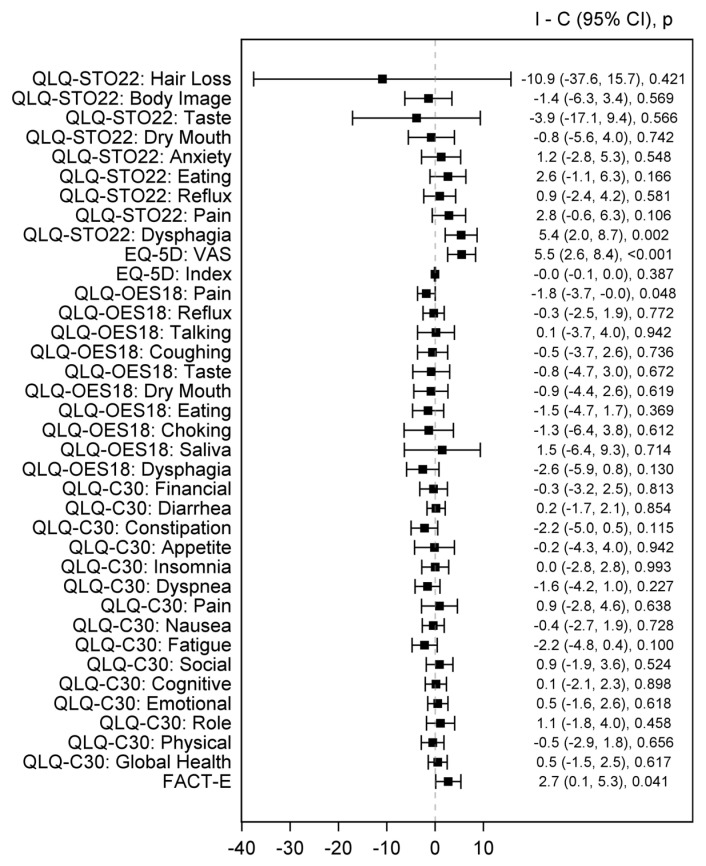
Forest plot of pooled estimates comparing ICI (combination with chemotherapy or combination ICI or ICI monotherapy) arm and CT arms. Black square indicates SMD and error bars indicate a confidence interval of 95%. The square size indicates the proportional weight of the study on the combined SMD.

The pooled analysis favored the ICI arm (both monotherapy and combination regimens) over the chemotherapy arm in terms of the Functional Assessment of Cancer Therapy—Esophageal (FACT-E; *n* = 2, Mean Difference [MD] 2.7 [95% 0.1, 5.3], *p* = 0.041), European Organization for Research and Treatment of Cancer (EORTC) Quality of Life Questionnaire—Esophageal Module (QLQ-OES18) pain scale (*n* = 3, MD −1.8 [95% CI −3.7, −0.0], *p* = 0.048), EuroQol-5D (EQ-5D) pain scale (*n* = 2, MD 5.5 [95% CI 2.6, 8.4], *p* < 0.001), and Quality of Life Questionnaire—Stomach Cancer Module (QLQ-STO22) dysphagia subscale (*n* = 2, MD 5.4 [95% CI 2.0, 8.7], *p* = 0.002). The pooled analyses were homogenous (Q *p* > 0.1, I2 = 0%). On the contrary, the pooled effect estimates showed a comparable EORTC QLQ-C30 Global Health (*p* = 0.617) and EQ-5D index (*p* = 0.367) between study arms (Figure 2).

QLQ-STO22 (Quality of Life Questionnaire-Stomach), EQ-5D (EuroQol 5 Dimension 5 Level), VAS (Visual analogue scale), QLQ-OES18 (Quality of Life Questionnaire—Esophageal cancer-specific module), QLQ-C30 (Core Quality of Life Questionnaire), FACT-E (Functional Assessment of Cancer Therapy—Esophageal).

**Figure 3:**
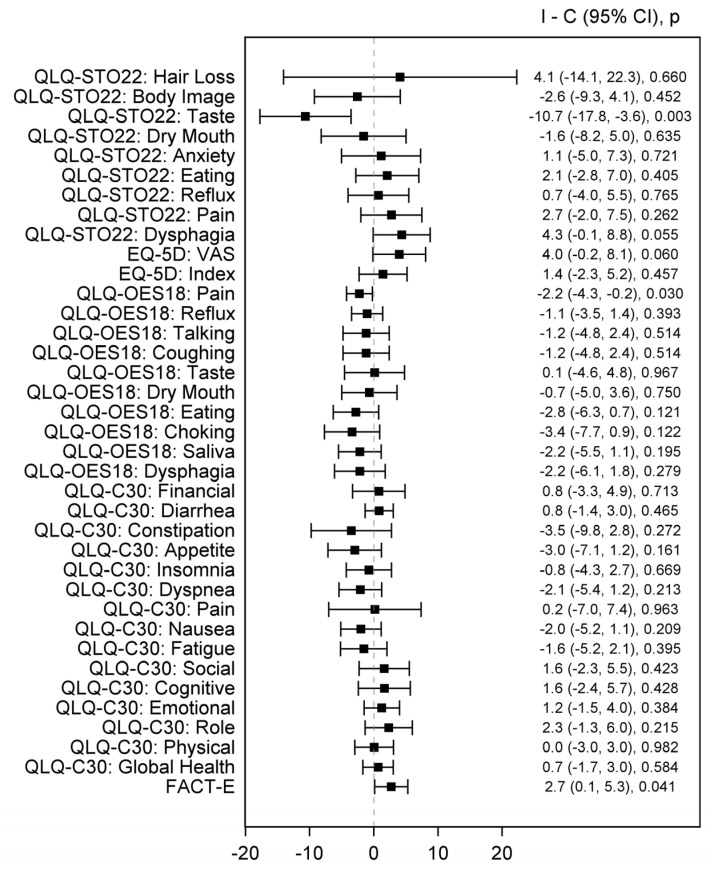
Forest plot of pooled estimates comparing combination therapy (ICI plus CT) and CT arms. Black square indicates SMD and error bars indicate a confidence interval of 95%. The square size indicates the proportional weight of the study on the combined SMD.

In the subgroup analysis comparing ICIs plus chemotherapy versus chemotherapy alone, the effect estimates favored the ICIs plus chemotherapy arm over the chemotherapy arm regarding FACT-E (*n* = 2, MD 2.7 [95% CI 0.1, 5.3], *p* = 0.041) and the EORTC QLQ-OES18 pain scale (*n* = 2 MD −2.2 [95% CI −4.3, −0.2], *p* = 0.030); the pooled analyses were homogenous (Q *p* > 0.1, I2 = 0%) (Figure 3).

QLQ-STO22 (Quality of Life Questionnaire-Stomach), EQ-5D (EuroQol 5 Dimension 5 Level), VAS (Visual analogue scale), QLQ-OES18 (Quality of Life Questionnaire—Esophageal cancer-specific module), QLQ-C30 (Core Quality of Life Questionnaire), FACT-E (Functional Assessment of Cancer Therapy—Esophageal).

**Figure 4:**
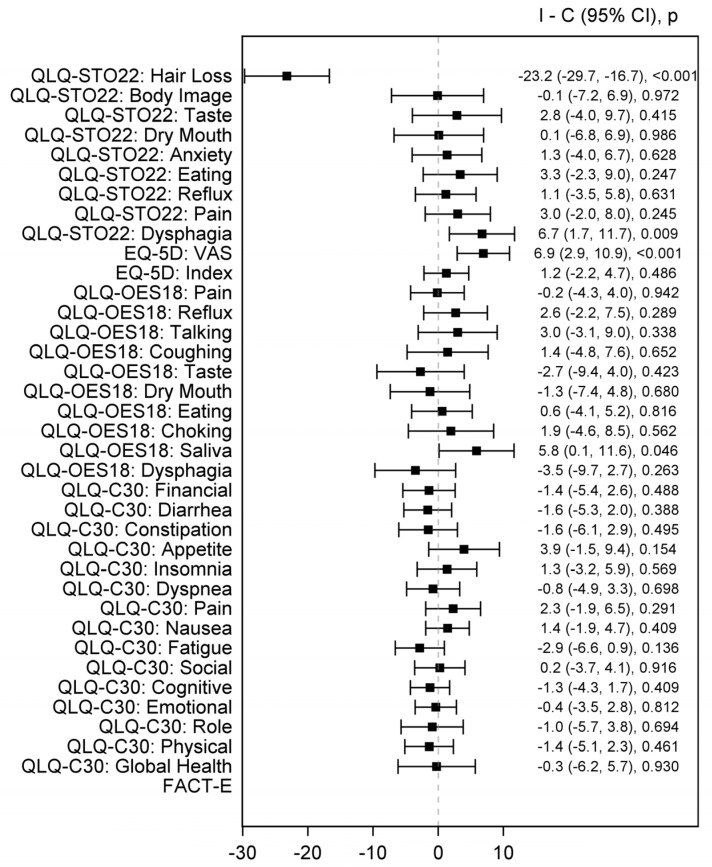
Forest plot of pooled estimates comparing ICI only (combination or monotherapy) arms and CT arms. Black square indicates SMD and error bars indicate a confidence interval of 95%. THE square size indicates the proportional weight of the study on the combined SMD.

Likewise, the effect estimates favored the ICI only arms over the chemotherapy arms regarding the QLQ-STO22 hair loss subscale [MD −23.2 (95% CI −29.7 to −16.7), *p* < 0.001], QLQ-STO22 dysphagia subscale [MD 6.7 (95% CI 1.7 to 11.7), *p* = 0.009], EQ-5D pain scale [MD 6.9 (95% CI 2.9 to 10.9), *p* < 0.001], and QLQ-OES18 saliva subscale [MD 5.8 (95% CI 0.1 to 11.6), *p* = 0.046) (Figure 4). There were no significant differences between the ICI monotherapy and the chemotherapy arms in other HRQoL scales and subscales (*p* > 0.05), which can be attributed to the small number of trials in this subgroup.

QLQ-STO22 (Quality of Life Questionnaire-Stomach), EQ-5D (EuroQol 5 Dimension 5 Level), VAS (Visual analogue scale), QLQ-OES18 (Quality of Life Questionnaire—Esophageal cancer-specific module), QLQ-C30 (Core Quality of Life Questionnaire), FACT-E (Functional Assessment of Cancer Therapy—Esophageal).

**Figure 5:**
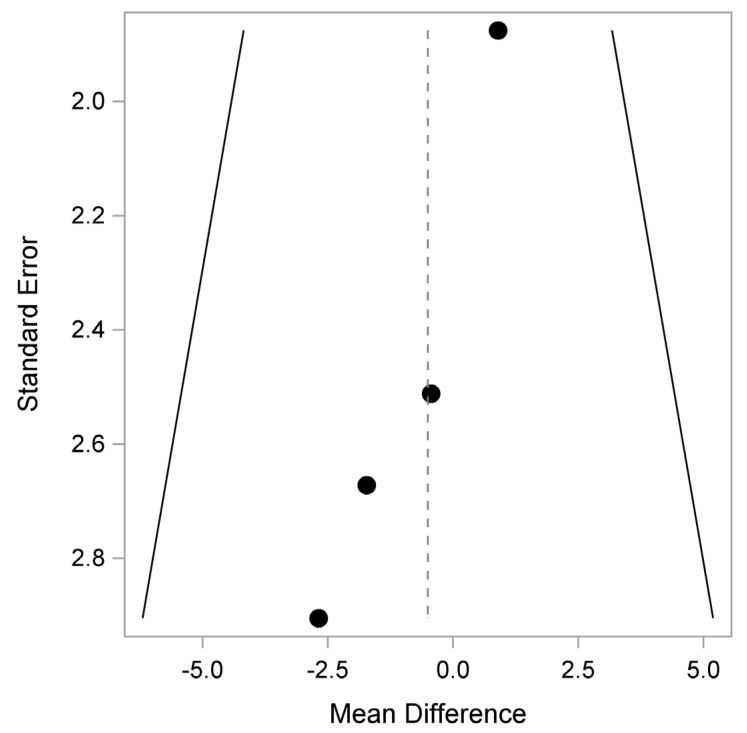
QLQ-C30: Physical (Quality of Life Questionnaire). Plot of the observed mean difference in the QLQ-C30 Physical Function score (x-axis) versus the corresponding standard error (SE; y-axis). Egger’s test (*p* = 0.037) indicates that there is a potential pattern, where the mean difference shifts with study size.

**Figure 6:**
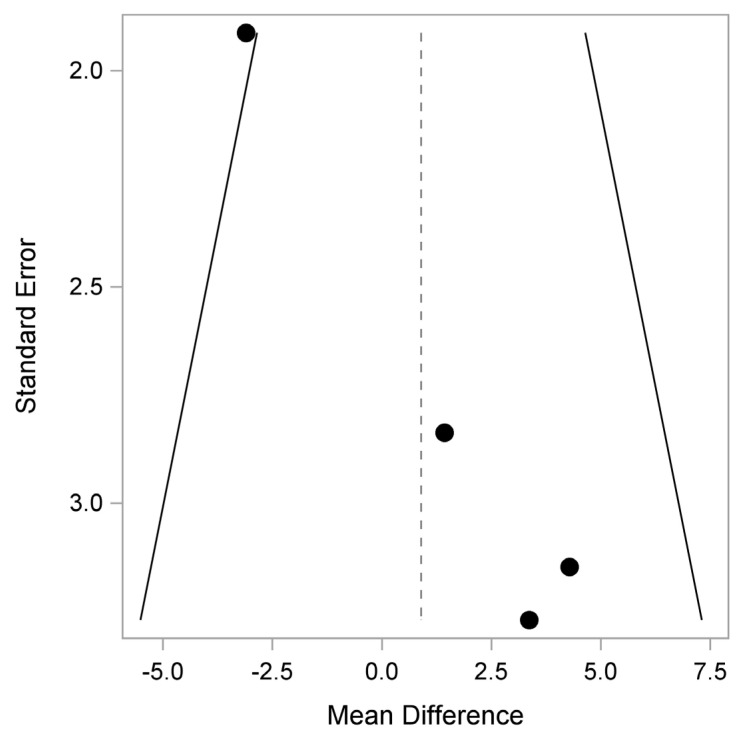
QLQ-C30: Pain (Quality of Life Questionnaire). Plot of the observed mean difference in the QLQ-C30 Pain score (x-axis) versus the corresponding SE (y-axis). Egger’s test (*p* = 0.013) indicates that there is a potential pattern, where the mean difference shifts with study size. Additionally, one study exhibits a mean difference outside the expected range given its sample size.

**Figure 7:**
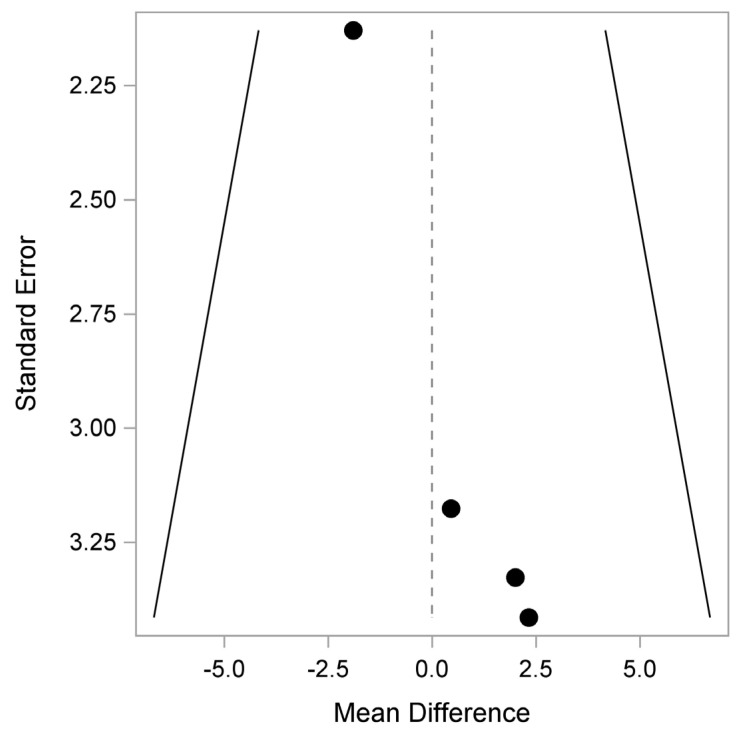
QLQ-C30: Insomnia (Quality of Life Questionnaire). Plot of the observed mean difference in the QLQ-C30 Insomnia score (x-axis) versus the corresponding SE (y-axis). Egger’s test (*p* = 0.026) indicates that there is a potential pattern, where the mean difference shifts with study size.

In terms of bias, there may be some in QLQ-C30 Physical, Pain and Insomnia, as shown in Figure 5, Figure 6 and Figure 7.

## 4. Discussion

In this study, we demonstrated the positive impact of ICIs on the HRQoL of patients diagnosed with GECs. Specifically, we found that ICIs, used as monotherapy or combined with chemotherapy, significantly improved patients’ HRQoL and managed disease-specific symptoms such as pain and dysphagia more effectively than chemotherapy alone. Our findings support the incorporation of ICIs into first-line treatment regimens for GECs, as they present similar or superior efficacy and enhance patients’ HRQoL.

To our knowledge, the current study represents the first to include a meta-analysis demonstrating that ICIs, or a combination of ICIs and chemotherapy, significantly improve the HRQoL of patients with GECs [30]. Notably, prior work suggests a significant association between impaired HRQoL and survival in patients with GEC [31], yet further studies with extended follow-up periods are required to fully understand the mechanistic linkage(s) among treatment with ICIs, improved survival, and enhanced HRQoL.

The findings from our current study demonstrate that ICIs, or a combination of ICIs and chemotherapy, may help to improve patient-reported symptoms. Specifically, we found that the studies suggested a significant improvement in patients’ pain scores with ICI treatment based on our pooled analysis. The observed improvement in pain management with ICIs may be attributed to the enhanced response rates, resulting in tumor shrinkage and less pain due to the cancer [32]. The differences in pain favoring ICIs may relate to the distinct mechanism of action compared to chemotherapy. ICIs primarily target specific immune checkpoints, resulting in fewer direct cytotoxic effects on healthy cells and organs and potentially leading to reduced pain levels [33]. Aligning with our findings, a meta-analysis of five RCTs found that PD-1/PD-L1 inhibitors led to longer times to the first clinically significant deterioration in HRQoL scores, as well as improvements in physical function and pain in patients with lung cancer [34]. A recent subgroup analysis of the CheckMate 649 trial showed more significant improvements in the FACT-Ga total, GaCS, and EQ-5D visual analog scale in patients receiving a combination of ICIs and chemotherapy than in patients receiving chemotherapy, with a reduction in HRQoL deterioration [35]. We also found that patients with GEC who received ICIs had significantly improved dysphagia scores compared to those who received chemotherapy. The reduced direct cytotoxic effects of ICIs on healthy cells and organs might result in decreased inflammation and swelling in the esophagus and stomach, potentially facilitating better swallowing [33], while also helping to avoid side effects such as nausea and vomiting, which frequently accompany chemotherapy. These findings for enhanced patient-reported symptoms with ICI treatment represent important patient-centered outcomes for individuals receiving treatment for GECs. Nevertheless, additional work is needed to delineate the underlying mechanisms further.

Debate exists regarding the need for all patients with gastroesophageal adenocarcinoma to receive immunotherapy in the frontline setting. High-quality evidence exists regarding the benefits offered to those with HER-2-negative adenocarcinoma with a higher PD-L1 expression [16]. An individual patient-level data analysis may show whether PD-L1 expression can also be used as a biomarker for HRQoL-related outcomes in GEC patients treated with immunotherapy. Similarly, further research may identify other clinical or molecular biomarkers predictive of patient-reported outcomes.

This study has several limitations that merit discussion. First, the number of included RCTs was relatively small, particularly in the ICI monotherapy subgroup. Second, the variability in HRQoL tools used across the included studies may have limited our ability to comprehensively assess the impact of ICIs on HRQoL. Third, our analysis focused on aggregate HRQoL measures and did not explore individual patient factors, such as age, gender/sex, or tumor stage, which may influence these scores. Fourth, even though the included RCTs utilized validate tools, recall bias could not be entirely eliminated. We could not assess the risk of bias on the quality of life of individual studies. Lastly, the follow-up duration in the included studies could have been longer, as it may not have captured the long-term impact of ICIs on HRQoL.

In conclusion, our study provides innovative findings regarding the significant improvement in HRQoL among patients with GEC who received treatment with ICIs. We demonstrated the notable potential of ICIs, alone or combined with chemotherapy, to enhance the clinical efficacy of GEC treatment and substantially improve patients’ HRQoL outcomes. Further research is needed to discover biomarkers, explore the long-term effects and potential mechanisms of ICIs on HRQoL, and identify patients who may benefit most from ICIs.

## Figures and Tables

**Table 1:** Summary characteristics of the included studies.

Trial	Design	Population	ICI Regimen	CT Regimen	No.	Primary Endpoint	QoL Measure
CHECKMATE 648	Phase 3 single-blinded RCT	Previously untreated, unresectable advanced, recurrent, or metastatic esophageal squamous cell carcinoma	Nivolumab + Chemotherapy; Nivolumab + ipilimumab	Fluorouracil + cisplatin	970	PFS, OS	FACT-E
ESCORT-1st	Phase 3 double-blinded RCT	Previously untreated advanced or metastatic esophageal squamous cell carcinoma	Camrelizumab + Chemotherapy	Paclitaxel + cisplatin	596	PFS, OS	QLQ-C30 and QLQ-OES18
KEYNOTE 062	Phase 3 single-blinded RCT	Previously untreated advanced gastric/gastroesophageal junction adenocarcinoma	Pembrolizumab	5-Fluorouracil or capecitabine + cisplatin	495	PFS, OS	QLQ-C30, QLQ-STO22
KEYNOTE 590	Phase 3 double-blinded RCT	Previously untreated locally advanced/unresectable or metastatic adenocarcinoma or esophageal squamous cell carcinoma or Siewert type 1 esophagogastric junction adenocarcinoma	Pembrolizumab + Chemotherapy	5-Fluorouracil + cisplatin	711	PFS, OS	QLQ-C30, QLQ-OES18, and EQ-5D-5L
KEYNOTE 181	Phase 3 open-label RCT	Previously treated advanced esophageal squamous cell carcinoma	Pembrolizumab	Paclitaxel, docetaxel, or irinotecan.	404	OS	QLQ-C30, OES18, and EQ-5D.
KEYNOTE 061	Phase 3 open-label RCT	Previously treated advanced gastric/gastroesophageal junction adenocarcinoma	Pembrolizumab	Paclitaxel	371	PFS, OS	QLQ-C30, QLQ-STO22, and EQ-5D-3L
ORIENT-15	Phase 3 double-blinded RCT	Previously untreated advanced or metastatic esophageal squamous cell carcinoma	Sintilimab + Chemotherapy	5-Fluorouracil or capecitabine + cisplatin	659	OS	EQ-5D-5L
ATTRACTION-3	Phase 3 open-label RCT	Previously treated advanced esophageal squamous cell carcinoma	Nivolumab	Paclitaxel or docetaxel	419	OS	EQ-5D-5L
ORIENT-16	Phase 3 double-blinded RCT	Previously untreated advanced gastric/gastroesophageal junction adenocarcinoma	Sintilimab + Chemotherapy	Oxaliplatin + capecitabine	650	OS	EQ-5D-5L
CheckMate 649	Phase 3 open-label RCT	Previously untreated, unresectable, non-HER2-positive gastric, gastro-esophageal junction, or esophageal adenocarcinoma,	Nivolumab + Chemotherapy	Capecitabine and oxaliplatin or leucovorin, fluorouracil, and oxaliplatin	1581	PFS, OS	FACT-G, EQ-5D U I UK set [Not extracted], EQ-5D VAS
ATTRACTION 4	Phase 3 double-blinded RCT	Previously untreated, unresectable, non-HER2-positive gastric or gastro-esophageal junction	Nivolumab + Chemotherapy	Oxaliplatin + capecitabine	724	PFS, OS	FACT-G, EQ-5D 3L

QoL (quality of Life), RCT (randomized controlled trial), PFS (progression-free survival), OS (overall survival), ICI (immune checkpoint inhibitor), CT (chemotherapy), QLQ-STO22 (Quality of Life Questionnaire—Stomach), EQ-5D (EuroQol 5 Dimension 5 Level), VAS (visual analogue scale), QLQ-OES18 (Quality of Life Questionnaire—Esophageal cancer-specific module), QLQ-C30 (Core Quality of Life Questionnaire), FACT-E (Functional Assessment of Cancer Therapy—Esophageal), FACT-G (functional assessment of cancer therapy—Stomach).

**Table 2:** MeSH keywords used as part of our search strategy.

Gastric Cancer	Gastroesophageal Cancer	Anti-PDL1	Gastric Adenocarcinoma	PD-1 Inhibitor
Avelumab	Gastric squamous cell carcinoma	PDL-1 Inhibitor	Esophageal adenocarcinoma	Nivolumab
Atezolizumab	Esophageal cancer	Durvalumab	Immune checkpoint inhibitor	Keytruda
Imfinzi	Bavencio	Tecentriq	Esophageal squamous cell carcinoma	Pembrolizumab

**Table 3:** Baseline characteristics and survival outcomes of the included studies.

Trial	Arms	No.	Age, Median (Range)	Male Sex, No. (%)	ECOG ≥ 1, No. (%)	Histologic Type, No. (%)		PD-L1 Expression ≥ 1%, No. (%)	Metastatic, No. (%)	Number with Metastases ≥ 2, No. (%)	Esophagectomy/Gastrectomy	mPFS	mOS
						SSC	Adeno						
CHECKMATE 648	Nivolumab + Chemotherapy	321	64 (40–90)	253 (79)	171 (53)	311 (97)	-	158 (49)	184 (57)	163 (51)	0	5.8	13.2
	Nivolumab + ipilimumab	325	64 (40–90)	269 (83)	174 (54)	322 (99)	-	158 (49)	196 (60)	165 (51)	0	2.9	12.7
	Chemotherapy	324	64 (26–81)	275 (85)	170 (52)	318 (98)	-	157 (48)	187 (58)	166 (51)	0	5.6	10.7
ESCORT-1st	Camrelizumab + Chemotherapy	298	62 (56–66)	260 (87.2)	227 (76.2)	298 (100)	-	166 (55.7)	298 (100)	160 (53.7)	116 (38.9)	6.9	15.3
	Chemotherapy	298	62 (56–67)	263 (88.3)	232 (77.9)	298 (100)	-	163 (54.7)	298 (100)	141 (47.3)	95 (31.9)	5.6	12
KEYNOTE 062	Pembrolizumab	239	-	-	-	-	-	-	-	-	-	-	-
	Chemotherapy	234	-	-	-	-	-	-	-	-	-	-	-
KEYNOTE 590	Pembrolizumab + Chemotherapy	356	-	-	-	-	-	-	-	-	-	-	-
	Chemotherapy	355	-	-	-	-	-	-	-	-	-	-	-
KEYNOTE 181	Pembrolizumab	217	-	-	-	-	-	-	-	-	-	-	-
	Chemotherapy	188	-	-	-	-	-	-	-	-	-	-	-
KEYNOTE 061	Pembrolizumab	188	-	-	-	-	-	-	-	-	-	-	-
	Chemotherapy	183	-	-	-	-	-	-	-	-	-	-	-
ORIENT-15	Sintilimab + Chemotherapy	327	63 (57–67)	279 (85)	250 (76)	327 (100)	-	174 (53)	285 (87)	-	96 (29)	7.2	16.7
	Chemotherapy	332	63 (56–67)	288 (87)	251 (76)	332 (100)	-	188 (57)	287 (86)	-	113 (34)	5.7	12.5
ATTRACTION-3	Nivolumab	210	64 (57–69)	179 (85)	109 (52)	210 (100)	-	101 (48)	94 (88)	121 (58)	111 (53)	1.7	10.9
	Chemotherapy	209	67 (57–72)	185 (89)	102 (49)	209 (100)	-	102 (49)	100 (83)	118 (56)	94 (45)	3.4	8.4
ORIENT-16	Sintilimab + Chemotherapy	327	-	-	-	-	-	-	-	-	-	-	-
	Chemotherapy	323	-	-	-	-	-	-	-	-	-	-	-
CheckMate 649	Nivolumab + Chemotherapy	789	62 (54–69)	540 (68)	463 (59)	-	-	126 (16)	757 (96)	602 (76)	-	7.7	13.8
	Chemotherapy	792	61 (53–68)	560 (71)	455 (57)	-	-	127 (16)	756 (95)	583 (74)	-	6.9	11.6
ATTRACTION 4	Nivolumab + Chemotherapy	362	64 (25–86)	253 (70)	167 (46)	-	-	58 (16)	-	254 (70)	105 (29)	10.5	17.5
	Chemotherapy	362	65 (27–89)	270 (75)	168 (46)	-	-	56 (15)	-	257 (71)	104 (29)	8.3	17.2

## Data Availability

The original contributions presented in the study are included in the article, further inquiries can be directed to the corresponding author/s.
